# Factors Associated with Malaria Infection among Head Porters in Agbogbloshie Market in the Greater Accra Region of Ghana

**DOI:** 10.1155/2020/8822165

**Published:** 2020-10-29

**Authors:** Patience Kwofie, John Tetteh, Reindorf Elijah Akakpo, Bismark Sarfo

**Affiliations:** ^1^National Cardiothoracic Centre, Korle-Bu Teaching Hospital, Accra, Ghana; ^2^Department of Epidemiology and Disease Control, School of Public Health, University of Ghana, Ghana; ^3^Department of Community Health, University of Ghana Medical School, College of Health Sciences, University of Ghana, Ghana

## Abstract

**Background:**

Head porters constitute the mobile population who are at an increasing risk of being infected with malaria. They move around the city to carry out their duties with no accommodation. Therefore, they sleep wherever they find themselves in the evening and do not benefit from most of the malaria intervention programs such as the use of long-lasting insecticide net. The inability to identify them because they are mobile means that they can continue to drive malaria transmission even if malaria in the general population is controlled.

**Objectives:**

This study assessed the factors associated with malaria infection among head porters in the Agbogbloshie market in the Greater Accra Region of Ghana.

**Method:**

A total of 218 head porters were recruited from the Agbogbloshie market, and blood samples were collected from participants to test for malaria parasite infection using Rapid Diagnostic Test (RDT) and microscopy and were interviewed using a closed-ended questionnaire. The data were analyzed using Stata version 15. Simple descriptive statistics, Pearson chi-square, and Multiple Logistic Regression were performed with significance set at <0.05.

**Result:**

The study revealed 12% (CI 95% = 8.2‐16.9) and 9.6% (CI 95% = 6.3‐14.4) infection of malaria using RDT and microscopy, respectively. *Plasmodium falciparum* (21/218) was the main parasite detected in all positive blood samples. Age and marital status are significant factors associated with malaria infection among head porters. Age group 40 years and above had 89% (AOR 0.11 (CI 95% = 0.01‐0.98)) reduced odds of getting malaria compared to those below 20 years, while those who are single are 3.52 times more likely to be infected with malaria compared with those who are married (AOR (95%CI) = 3.52 (1.13‐10.92)).

**Conclusion:**

This study concludes that the increasing age of head porters significantly decreased the probability of malaria infection, while head porters who are single have greater odds of being infected with malaria. Age and marital status are important factors to be considered for malaria intervention programs in head porters.

## 1. Introduction

The World Health Organization (WHO) report as at December 2019, indicated an estimated 228 million cases of malaria globally, out of which 405,000 deaths occurred mostly in African countries [1]. Various intervention efforts have been put in place over the decade to reduce malaria transmission, and this has resulted in the decline of the disease globally.

In Africa, there has been a long-term decline in the prevalence of *Plasmodium falciparum* malaria from 40% to 24% in the period 1900-1929 and 2010-2015, respectively [2].

Ghana is among the 15 countries with malaria burden in the world, accounting for 4% of the global cases and 7% of all malaria cases in West Africa [2, 3].

Testing of suspected malaria cases in Ghana has gone up significantly from 39% to 78% from 2013 to 2016, and this has increased laboratory-confirmed cases from 143 per 1,000 population to 166 per 1,000 population, leading to a decline in malaria attributable mortality from 19% in 2010 to 4.2% in 2016 [4].

Over the past decade, Ghana has made some progress in reducing malaria morbidity and mortality, and this resulted in decreased dominance of the various *Plasmodium* species [5].

Notwithstanding this achievement, there is a high prevalence of malaria in some districts in Ghana especially in the rural areas compared with the urban centers. The 2016 multiple indicator survey shows a 28% prevalence of malaria in the rural areas compared with 11% in the urban areas [5, 6]. The high prevalence of malaria in such areas could be attributed to operational challenges in the implementation of the various intervention programs.

The human population movement (HPM) has been described as a major challenge facing malaria control and elimination programs [7]. Mobile populations from highly endemic malaria regions serve as a high risk in introducing malaria to the malaria-free areas thereby compromising the effort put in place to eradicate it [7, 8]. An understanding of the epidemiology of malaria through multiple locations in association with population movements is required to make the implementation of malaria programs more meaningful, practicable, and impactful [8].

Human population growth is one of the important factors in malaria transmission, and for any intervention program to be successful, this factor must be taken into consideration [9, 10]. Urbanization and rapid population growth have been on the rise in many sub-Saharan African regions including Ghana with an increased rate of internal migration by people such as head porters seeking menial jobs in the cities and towns [11]. These people do not have accommodation, and they move about and congregate at shops and other open spaces in commercial towns and cities [11]. Therefore, they sleep wherever they find themselves in the evening and do not benefit from protection against malaria such as the use of long-lasting insecticide net and indoor residual spraying [9, 10]. This group of population is highly vulnerable to malaria because of barriers to access basic and quality health care services [10, 11]. Infection of malaria in this vulnerable population could serve as a source of residual transmission which may impact all the efforts put in place to control the disease. Therefore, it is important to understand the factors associated with malaria transmission in this mobile population to help develop targeted interventions for the national malaria control program. The absence of known drivers of malaria in this special population means that malaria infection in this group can continue to drive the transmission even if malaria in the general population is controlled. This study assessed the factors associated with malaria infection among head porters in the Agbogbloshie market in the Greater Accra Region of Ghana.

## 2. Methods

### 2.1. Study Site

**Figure 1:**
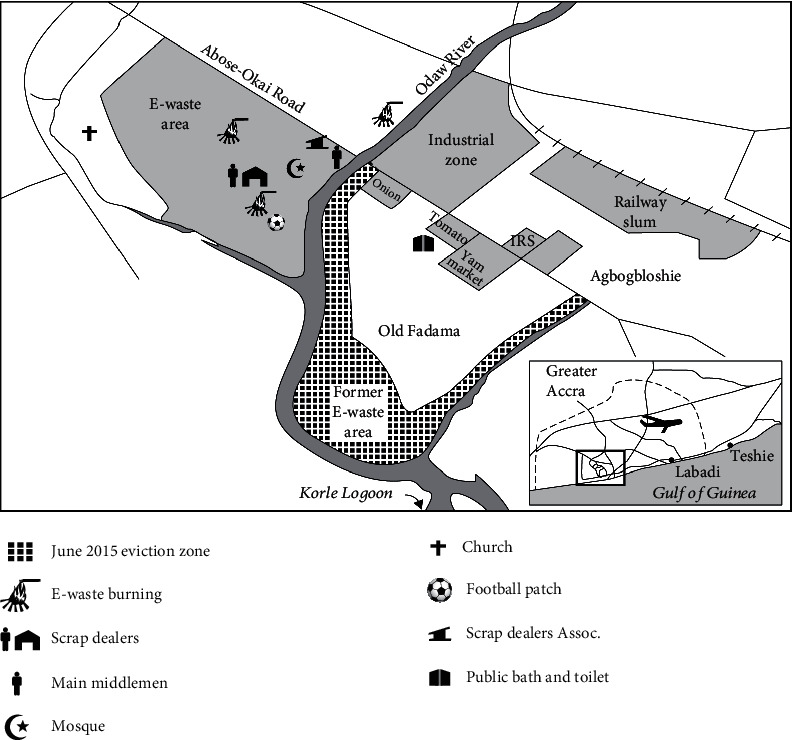
Catchment area of Agbogbloshie in the Greater Accra Region. Source: Adopted from Daum et al. [13].

The study was conducted in the Agbogbloshie market in the Accra Metropolis in the Greater Accra region of Ghana. Agbogbloshie is located in the Ashiedu Keteke, a submetro of the Accra Metropolis in the Greater Accra Region. The 2010 population census report indicated that the population of Agbogbloshie area is about 8,305 with females constituting 54% while males form 46% [12]. The market area is noted as the dumping ground for electronic waste. In this area, electronic and electrical waste as well as other metals from vehicles are dismantled and traded as scraps. There is a river called Odaw, which surrounds the two sides of the market, and this river runs through Accra and flows into the Gulf of Guinea (Figure 1, [13]). The river is heavily polluted with electronic and other scrap materials and serves as additional breeding grounds for mosquitoes in the area. The Agbogbloshie market itself is noted for agricultural produce including tomatoes, beans, maize, onions, and especially yams. Traders bring these farm produce from all over the country to sell in this market, and most people residing in the capital city of Accra buy their food items from this market. Another informal settlement that is adjacent to Agbogbloshie is Old Fadama. These two communities constitute one of the biggest urban slums in Ghana.

### 2.2. Study Participants

The study population was made up of head porters who reside at the Agbogbloshie market. Most of these head porters are internal migrants who travelled from rural areas especially from the northern regions of Ghana to the capital city, Accra, to search for menial jobs. The exact total population of head porters in Accra including Agbogbloshie is unknown because they are always missed out during household or institutional surveys. However, the estimated population of head porters in Accra ranges from 2,300 to 160,000 [14, 15]. The main work that they do is to carry goods especially food items for shoppers who come to the Agbogbloshie market, and as a result, they move a lot within the market and also from one market to another within the Agbogbloshie catchment area. The overwhelming majority (anecdotal evidence) of the head porters in the Agbogbloshie market and indeed in the capital city of Accra are females. They charge a fee for providing such services, and that is how they earn their income. Because the head porters move from other parts of the country especially the north to this market area as internal economic migrants, they do not have any accommodation. As such, almost all of them sleep in the open verandas in front of people's shops, and some sleep in wooden kiosks in the market, exposing themselves to the bites of mosquitoes. In a similar study carried out among head porters in Madina, a suburb of Greater Accra, a malaria prevalence of 15.1% was reported [10]. All the head porters who agreed to participate were included in the study.

### 2.3. Study Design and Sample Size Estimation

A cross-sectional design was used in this study. The sample size for the study was determined using the formula  *N* = (*Z*^2^*p*(1 − *p*))/*d*^2^ where, *Z* is *z* statistic for a 95% level of confidence, *p* is expected prevalence or proportion in the population based on previous studies (15.1%) [10], and *d* is the margin of error or precision (0.05).

Using the following formula: *N* = (1.96^2^ × 0.15(1 − 0.15))/(0.05^2^) = 195.9.

With the addition of the 10% nonresponse rate, the total minimum sample size for the study was 215.

## 3. Variables

The outcome variable for the study is malaria infection which was tested by RDT and microscopy. Independent variables included age, sex, education, marital status, malaria awareness, causes, transmission, and preventive measures.

### 3.1. Data Collection Technique

Agbogbloshie market was selected because it is highly populated with head porters, who are also called “kayayie” in the local parlance. The National Office for the Association of head porters in Ghana is also located in the Agbogbloshie market. Information regarding the study population was taken from the President of the National head porters association in Ghana to help identify the location and time from which they can be contacted. The participants were enrolled through a simple random sampling method where the participants were asked to pick at random a piece of paper with “yes” or “no” inscriptions from a bowl. Those who chose a paper with “yes” were enrolled while those who chose “no” were not enrolled. Head porters 18 years and above were included in the study. During the enrolment, a durbar was organised by the President of the association to assemble all the head porters available on three different occasions. In the first durbar, all the head porters who were enrolled were given different unique identification codes. These first batch of participants were asked not to come again during the second and third durbars. Those who came during the second durbar and were enrolled, were also given different unique identification codes, and were asked not to participate in the subsequent durbar. This same procedure was repeated for the third time before the required sample size was obtained. Written consent was taken from the participants who were eligible for the study. Those who did not consent were excluded. A closed-ended interviewer questionnaire was used to obtain information about the factors associated with malaria transmission. The interviewer questionnaire was pretested among the head porters who reside at the Kaneshie market within the Greater Accra Metropolis.

For each participant, after being interviewed, the blood sample was taken and tested for the presence of malaria parasites.

Finger prick blood samples were collected from participants to test for malaria using the Rapid Diagnostic Test (RDT) and microscopy. The RDT malaria screening was performed using the CareStart™ Malaria test kit (Histidine Rich Protein 2) HRP2 (Pf) (Access Bio, INC, 65 Clyde Road Suite A Somerset NJ 08873 USA) following the manufacturer's instruction. This kit was recommended by the Ghana National Malaria Control Program (NMCP). Finger prick blood samples from participants were also used to make thick smears, and examined for malaria parasites after staining with 5% Giemsa. Blood smears were read by two trained microscopists using the World Health Organization (WHO, 2010 [16]) standard procedures [17]. Malaria parasitaemia was defined by the presence of the asexual form of *Plasmodium* by microscopic examination of the thick blood smears stained with Giemsa.

Parasite density per microliter of blood was estimated using the formula  (total WBC per *μ*l blood/#of WBC counted) × #of parasites counted.

Positive malaria cases were managed with artemether 20 mg+lumefantrine 120 under a healthcare provider.

### 3.2. Ethical Clearance

Ethical clearance was sought from the Ghana Health Service Ethics Review Committee with approval number GHS-ERC164/412/17. Permission was obtained from the President of the National head porters association in Ghana, and also, a written consent was taken from the participants. All those who tested positive for malaria were treated with artemether 20 mg+lumefantrine 120 under a healthcare provider.

### 3.3. Data Analysis

Data were entered into Excel and imported into Stata version 15.0 (College Station, TX 77845, USA) for processing and analysis. The outcome variable is malaria infection. Two approaches of analysis were carried out, which involved descriptive and inferential statistics. Descriptive analysis was performed by running frequencies on all categorical variables and mean values for discrete variables. To assess the significant difference between the RDT and microscopy malaria prevalence, a 95% confidence interval error bar chart was adopted. Interrater agreement between the two malaria estimates performed by RDT testing and by microscopy was further assessed using the kappa statistics. Cohen's kappa statistics were calculated using the estimates of the observed and expected agreement between RDT and microscopy (two-by-two table). Inferential analysis was performed by cross-tabulations to assess the Pearson chi-square test of independence. Univariate and multivariate binary logistic regression analyses were performed separately. During the initial univariate analytical procedure, the backward selection model was adopted to select potential predictors in the study. A *p* value of <0.05 was considered to be statistically significant at 95% confidence interval.

## 4. Results

A total of 218 participants (over 100% response rate) were recruited at the Agbogbloshie market for the study. The age ranged between 17 years and 65 years with a mean (SD) of 29.5 (±10.32) years. The majority of the respondents were females (99.1%) with more of the head porters within the age group 20-29 years (41.7%). Also, the head porters who were married were more (51.8%), and those with no formal education were the most involved (67.9%) (Table 1).

**Figure 2:**
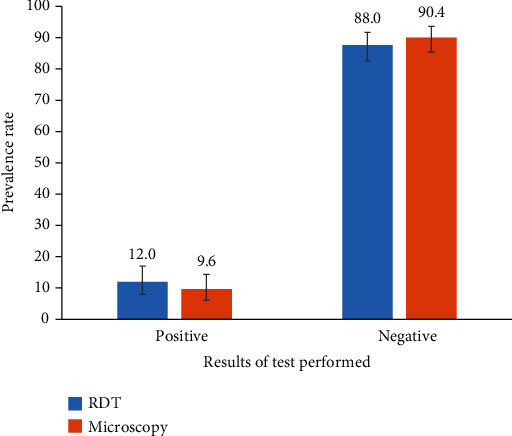
Prevalence of malaria using RDT and microscopy.

The presence of malaria infection in participants using RDT and microscopy is presented in the Figure 2 below. RDT and microscopy recorded 12% (95%CI = 8.2‐16.9) and 9.6% (95%CI = 6.3‐14.4) malaria infection, respectively. The difference between the RDT and microscopy prevalence is not statistically significant (Figure 2).

Meanwhile, Cohen's kappa for the two tests (RDT and microscopy) was 0.21 while the observed and expected agreement was 84.9% and 80.7%, respectively.

Microscopy positive test results show that all parasites present in blood samples of the head porters were *Plasmodium falciparum* species. Most of the parasites density was below 200/*μ*l (71.4%) with 19.1% having a density of 400/*μ*l and above (Table 2).

The Pearson chi-square test showed that the prevalence of malaria among the head porters was statistically significant (*p* < 0.05) for “awareness of malaria”, “causes of malaria”, “transmission of malaria,” and “preventive knowledge of malaria” of the study variables (Table 3).

The adjusted regression analysis demonstrated that head porters within the age of 40 years and above have reduced odds (89%) of getting malaria by using the RDT test kit compared to head porters below the age of 20 years (AOR (95%CI) = 0.11 (0.01‐0.98)). Interestingly, head porters who are single were 3.52 times more likely to be infected with malaria compared with those who are married (AOR (95%CI) = 3.52 (1.13‐10.92)) (Table 4). The Hosmer-Lemeshow goodness of fit test for this regression was 14.36 and 0.157 for chi and *p* value, respectively.

## 5. Discussion

This study has demonstrated 12% and 9.6% of malaria infection among head porters using RDT and microscopy, respectively, and that age and marital status (being single) are the significant factors associated with this infection. *Plasmodium falciparum* was the main parasite detected in all positive blood examinations using microscopy. Most of the participants who tested positive have parasite density between 16 and 199 parasites/microliter.

Although age, marital status, awareness of malaria, causes of malaria, knowledge on malaria transmission, and malaria preventive knowledge had significant association with malaria infection, only age and marital status were observed to be statistically significantly associated with malaria infection after adjusting for study variables. The prevalence of 12% and 9.6% are relatively lower compared to what was reported in Bosomtwe-Atwima Kwawoma in Ghana a decade ago [18] and lower than the prevalence of 15.1% among hawkers in a recent study conducted in the Greater Accra Region [10]. The relatively lower prevalence identified in this study as compared to previous studies might be attributed to geographical differences and or local demographic factors [10, 19, 20]. Also, malaria control strategies adopted in these geographical locations could be a probable factor.

This study corroborates with a previous study which also observed *Plasmodium falciparum* as the main parasite responsible for malaria in head porters [10]. This prevalence is slightly higher as compared to what was reported by WHO that the main *Plasmodium* species accounting for 99.7% of all malaria cases in the WHO African region and causing malaria morbidity and early mortality in sub-Saharan African regions is *falciparum* [2]. Again, this study revealed a marginal difference between the RDT results and the microscopy, although was not statistically significant. Five *Plasmodium falciparum* infections were missed by microscopy but were detected by the RDT. In relation to this, a previous study also identified a similar result using RDT and microscopy which detected a prevalence of 27.4% and 21.6%, respectively [21]. Respondents may have been treated within the past two weeks for malaria infection, and this could explain the marginal disparity between the RDT and the microscopy results. Leslie and colleagues [21] discovered that microscopy had a lower operational sensitivity for detection of *Plasmodium falciparum* than the RDT, and these have been explained by WHO. The difference between RDT and microscopy results could also be due to low parasitemia in the tested samples [21, 22].

Age has been identified to be statistically associated with RDT malaria prevalence as indicated in this study. It has been established that age and host genetic factors are important determinants of malaria prevalence [21], and many researchers have shown that age is significantly associated with the risk of developing malaria [23–26]. This study demonstrated that the odds of being infected with malaria among the head porters decreases as their ages increase. Our findings further reveal that the age group 40 years and above had 89% reduced odds of getting malaria compared to those below 20 years. It has been established that increasing age coupled with the number of mosquito bites is associated with the acquisition of immunity to malaria [27]. In favorable malaria transmission areas like Accra, as people age, the probability of been bitten by mosquito severally increases, and this could confer immunity to malaria infection as demonstrated in this group of participants. This study however reported that majority of the head porters are below 40 years, and therefore, most of them will not benefit from the protective effect of age in this instance, and this must be a concern for intervention policies targeting this special group of economic migrants.

This study has also demonstrated that head porters who were not married are more likely to be infected with malaria compared with those who are married. Over 76% of the asymptomatic malaria infections were recorded among head porters who are single, and those who are single have greater odds of being infected with malaria. This finding is consistent with our previous study which reported that marital status is one of the demographic factors that affected malaria infection [11]. Marriage in African society is seen as an incubator for social support, but since these head porters do not live with their partners during the time that this study was conducted, it makes the reason for marital status as a factor that can predispose or protect against malaria infection very complex. But the import of this finding is that, in developing any malaria intervention targeting this group of people, the marital characteristic should be a factor that should be considered.

### 5.1. Study Limitation

The cross-sectional nature of the design of this study precludes the generalizability of the findings to other groups of people living in malaria-endemic regions. Social desirability could also have affected the responses of some of the study participants. The findings of this study are however very important for any malaria intervention targeting head porters in the Greater Accra Region of Ghana.

## 6. Conclusion

This study concludes that the increasing age of head porters significantly decreased the probability of malaria infection, while head porters who are single have greater odds of being infected with malaria. Age and marital status are important factors to be considered for malaria intervention programs in head porters.

## Figures and Tables

**Table 1:** Demographic characteristics of study participants.

Variable	Frequency *N* = 218	Percentage (%)
Sex		
Male	2	0.9
Female	216	99.1
Age		
Below 20	32	14.7
20-29	91	41.7
30-39	48	22
40-49	34	15.6
50 and above	13	6.0
Mean(SD)	29.5 (10.32)	
Marital status		
Single	95	43.6
Married	113	51.8
Divorced	7	3.2
Widowed	3	1.4
Educational level		
No formal education	148	67.9
Primary school	26	11.9
Junior high school and above	44	20.2

**Table 2:** Falciparum parasite density related to microscopy test.

Parasite density/*μ*l	Frequency	Percentage
16-199	15	71.4
200-399	2	9.5
400 and above	4	19.1

**Table 3:** Association between study variables of malaria positive test using RDT.

Variable	RDT test results		Pearson chi-square	
	Negative = 192	Positive = 26	Chi	*p* value
Awareness malaria			4.75	0.029
Yes	170 (88.54)	19 (73.08)		
No	22 (11.46)	7 (26.92)		
Cause of malaria			6.4	0.011
Yes	136 (70.83)	12 (46.15)		
No	56 (29.17)	14 (53.85)		
Knowledge on vector			0.6	0.441
Mosquito	129 (94.85)	11 (100.00)		
Others	7 (5.15)	0 (0.00)		
Knowledge on transmission			3.87	0.049
Mosquito bite	113 (58.85)	10 (38.46)		
Other	79 (41.15)	16 (61.54)		
Signs and symptoms			2.56	0.109
Yes	120 (62.50)	12 (46.15)		
No	72 (37.50)	14 (53.85)		
Preventive knowledge			8.52	0.004
Yes	130 (67.71)	10 (38.46)		
No	62 (32.29)	16 (61.54)		
Specific prev. measures			14	0.496
ITN and IDRS	41 (32.03)	5 (50.0)		
ITN	65 (50.78)	4 (40.00)		
IDRS	22 (17.19)	1 (10.00)		

**Table 4:** Factors associated with malaria among head porters using logistic regression analysis.

Variable	RDT test result		COR (95% CI)	AOR (95% CI)	*p* value
	Negative = 192	Positive = 26			
Age					0.001
Below 20	22 (11.46)	10 (38.46)	Ref		
20-29	80 (41.67)	5 (17.31)	0.30 (0.11-0.80)^∗^	0.40 (0.14-1.15)	
30-39	44 (22.92)	4 (15.38)	0.20 (0.06-0.71)^∗^	0.37 (0.09-1.62)	
40 and above	46 (23.96)	7 (19.85)	0.05 (0.00-0.40)^∗∗^	0.11 (0.01-0.98)^∗^	
Marital status					0.004
Married	107 (55.73)	6 (23.08)	Ref		
Single	85 (44.27)	20 (76.92)	4.20 (1.6-10.9)^∗∗^	3.52 (1.13-10.92)^∗^	
Educational level					
Junior high school above	39 (20.94)	5 (19.23)	Ref		
No formal education	129 (67.19)	19 (73.08)	1.15 (0.40-3.30)		
Primary school	24 (12.50)	2 (7.69)	0.65 (0.12-3.62)		
Awareness of malaria					
Yes	170 (88.54)	19 (73.08)	Ref		
No	22 (11.46)	7 (26.92)	2.84 (1.08-7.53)^∗^		
Cause of malaria					
Yes	136 (70.83)	12 (46.15)	Ref		
No	56 (29.17)	14 (53.85)	2.83 (1.23-6.51)^∗∗^		
Knowledge on organism					
Mosquito	129 (94.85)	11 (100.00)	Ref		
Others	7 (5.15)	0 (0.00)	1		
Transmission					
Mosquito bite	113 (58.85)	10 (38.46)	Ref		
Other	79 (41.15)	16 (61.54)	2.29 (0.99-5.31)		
Signs and symptoms					
Yes	120 (62.50)	12 (46.15)	Ref		
No	72 (37.50)	14 (53.85)	1.94 (0.85-4.43)		
Preventive knowledge					0.004
Yes	130 (67.71)	10 (38.46)	Ref		
No	62 (32.29)	16 (61.54)	3.35 (1.44-7.82)^∗∗^	0.62 (0.26-1.64)	
Specific prev. measures					
ITN and IDRS	41 (32.03)	5 (50.0)	Ref		
ITN	65 (50.78)	4 (40.00)	0.50 (0.13-1.99)		
IDRS	22 (17.19)	1 (10.00)	0.37 (0.04-3.39)		

^∗^ *p* value <0.05 and ^∗∗^*p* value ≤0.01.

## Data Availability

The datasets during and/or analyzed during the current study are available from the corresponding author on reasonable request.
